# Targeting Ovarian Cancer with IL-2 Cytokine/Antibody Complexes: A Summary and Recent Advances

**DOI:** 10.33696/immunology.3.122

**Published:** 2021

**Authors:** Yilun Deng, Ryan M Reyes, Chenghao Zhang, José Conejo-Garcia, Tyler J. Curiel

**Affiliations:** 1 Department of Medicine, University of Texas Health San Antonio, San Antonio, TX, USA; 2 South Texas Medical Scientist Training Program, University of Texas Health San Antonio, San Antonio, TX, USA; 3 Mays Family Cancer Center, University of Texas Health San Antonio, San Antonio, TX, USA; 4 Xiangya Medical School, Central South University, Changsha, Hunan, China; 5 H. Lee Moffitt Cancer Research Institute, Department of Immunology, Tampa, FL, USA; 6 Graduate School of Biomedical Sciences, University of Texas Health San Antonio, San Antonio, TX, USA

**Keywords:** Ovarian cancer, Immunotherapy, IL-2, Regulatory T cell, IL-2 receptor

## IL-2 and IL-2 Receptor

**Figure 1:**
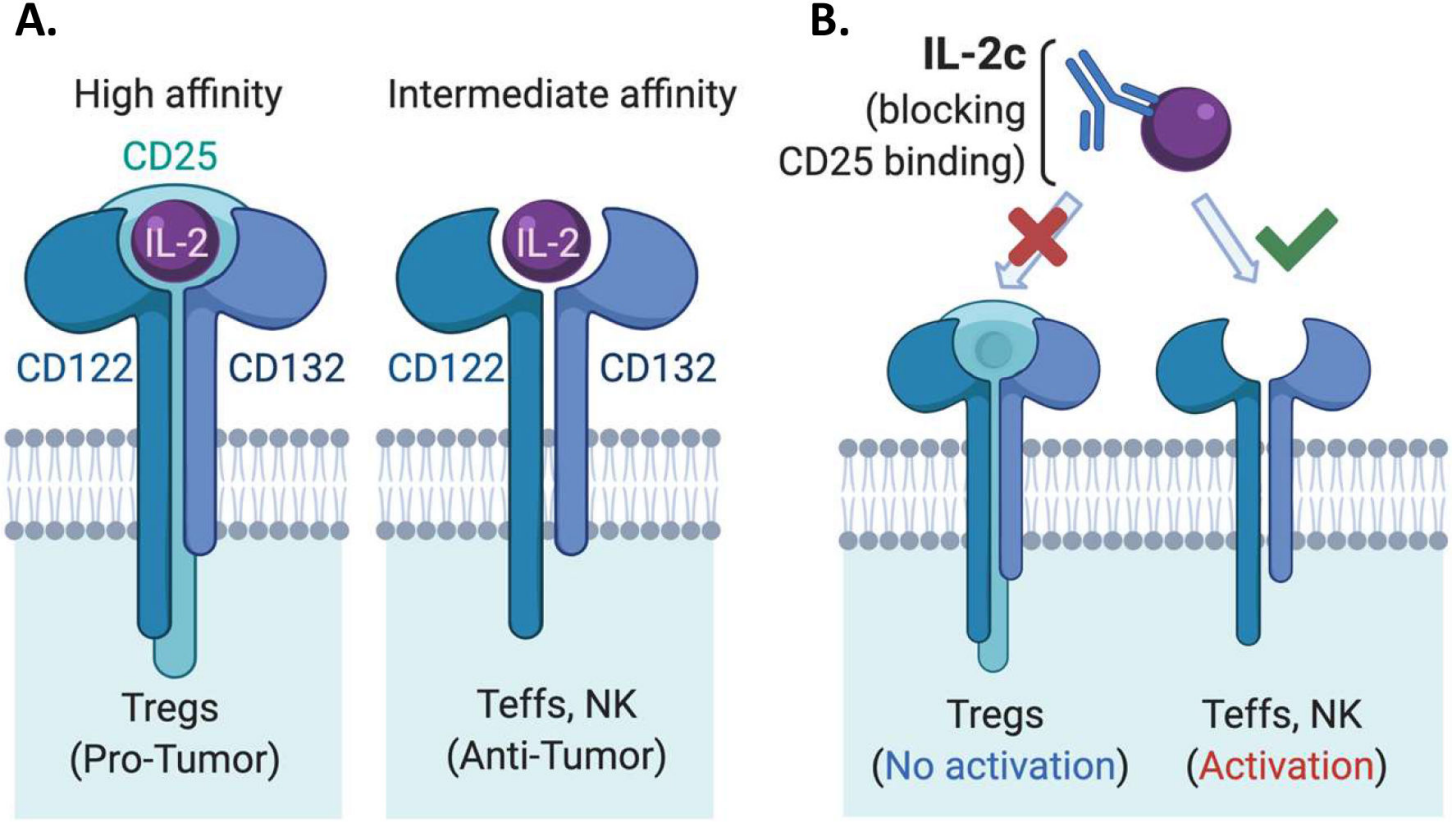
Graphic illustration of IL-2 receptor and IL-2 complex. **A.** High affinity and intermediate affinity IL-2 receptor structures. Tregs constitutively express high affinity IL-2R while effector T cells and NK cells express intermediate affinity IL-2R. **B.**Theoretical mechanism of action of IL-2c.

Interleukin (IL)-2 was first identified as a potent T cell growth factor in 1976 [1] and cloned in 1983 [2]. In the late 1990s, IL-2 gained further attention as the first immunotherapy demonstrating clinical efficacy against metastatic cancer, as high dose IL-2 was approved by the United States Food & Drug Administration (FDA) for the treatment of metastatic renal cell carcinoma in 1992 and metastatic melanoma in 1998 [3]. However, initial IL-2 immunotherapy treatment regimens led to severe and sometimes fatal toxicity in patients [4], significantly limiting its clinical application. Early treatment regimens were thought to require high doses of IL-2 for several reasons. IL-2 has a short *in vivo* half-life [5], requiring frequent dosing to maintain its biological effect of stimulating antitumor immunity. There are different forms of the IL-2 receptor (IL-2R) with different affinity for IL-2 [6] that express distinct cellular expression patterns (Figure 1A). Effector T cells (Teffs) and natural killer (NK) cells, linchpins of antitumor immunity, constitutively express intermediate-affinity IL-2Rs at baseline, which are dimeric receptors comprised of IL-2Rβ (CD122) and IL-2Rγ (common γ-chain or CD132) [6] subunits. Yet, a major immunosuppressive population in the tumor microenvironment (TME), regulatory T cells (Tregs) among other populations, express trimeric high-affinity IL-2Rs [6], which consist of IL-2Rβ, IL-2Rγ and IL-2Rα (CD25) subunits (Figure 1A). Therefore, unaltered IL-2 must be used at high doses for optimal Teff activation via intermediate affinity IL-2Rs as a trimeric IL-2R binds IL-2 with roughly 10-100-fold higher affinity than a dimeric IL-2R [5], rendering Tregs a great competitive advantage for IL-2 availability over Teffs in the TME.

## IL-2/anti-IL-2 mAb Complexes

To overcome limitations of native IL-2 in cancer treatment, many engineered forms or complexes of IL-2 have been developed in the past decade [7-21]. These approaches include PEGylated IL-2 agonists [18,19,22,23], IL-2 fusion proteins [12,23], IL-2 and anti-IL2 mAb complexes [7,24-26], IL-2 with the CD25 binding site genetically deleted [27] and IL-2 fused to CD25 [28] among others [23,29], aiming preferentially activate antitumor effector cells over Tregs, and in some cases also to prolong IL-2 *in vivo* half-life. These agents are in different stages of preclinical/clinical research, recently reviewed elsewhere [23]. In particular, several groups have designed IL-2 agents that preferentially signal to Teffs through intermediate-affinity IL-2Rs over Tregs, including Boyman and colleagues who described selective activation of immune populations by distinct IL-2/anti-IL-2 immune complexes in 2006 [7]. This group used an anti-IL-2 mAb clone JES6-5H4 (S4B6-like) that could sterically block the interaction between IL-2 and CD25 while conformationally stabilizing the IL-2:CD122 interaction, leading to preferential activation of CD122 expressing (in the intermediate affinity IL-2R) Teffs and NK cells over Tregs [16] (Figure 1B). This CD122-selective IL-2 complex (IL-2c) is the focus of this commentary as several groups have previously shown the efficacy of IL-2c in multiple murine cancer models including B16/F10 melanoma [9,10,30], pancreatic cancer model [8], BCL1 leukemia [9], TRAMP-C1 sarcoma [14], and orthotopic bladder cancer [31].

## IL-2c Treats an Immune Checkpoint Blockade Resistant Mouse Ovarian Cancer Model

**Figure 2:**
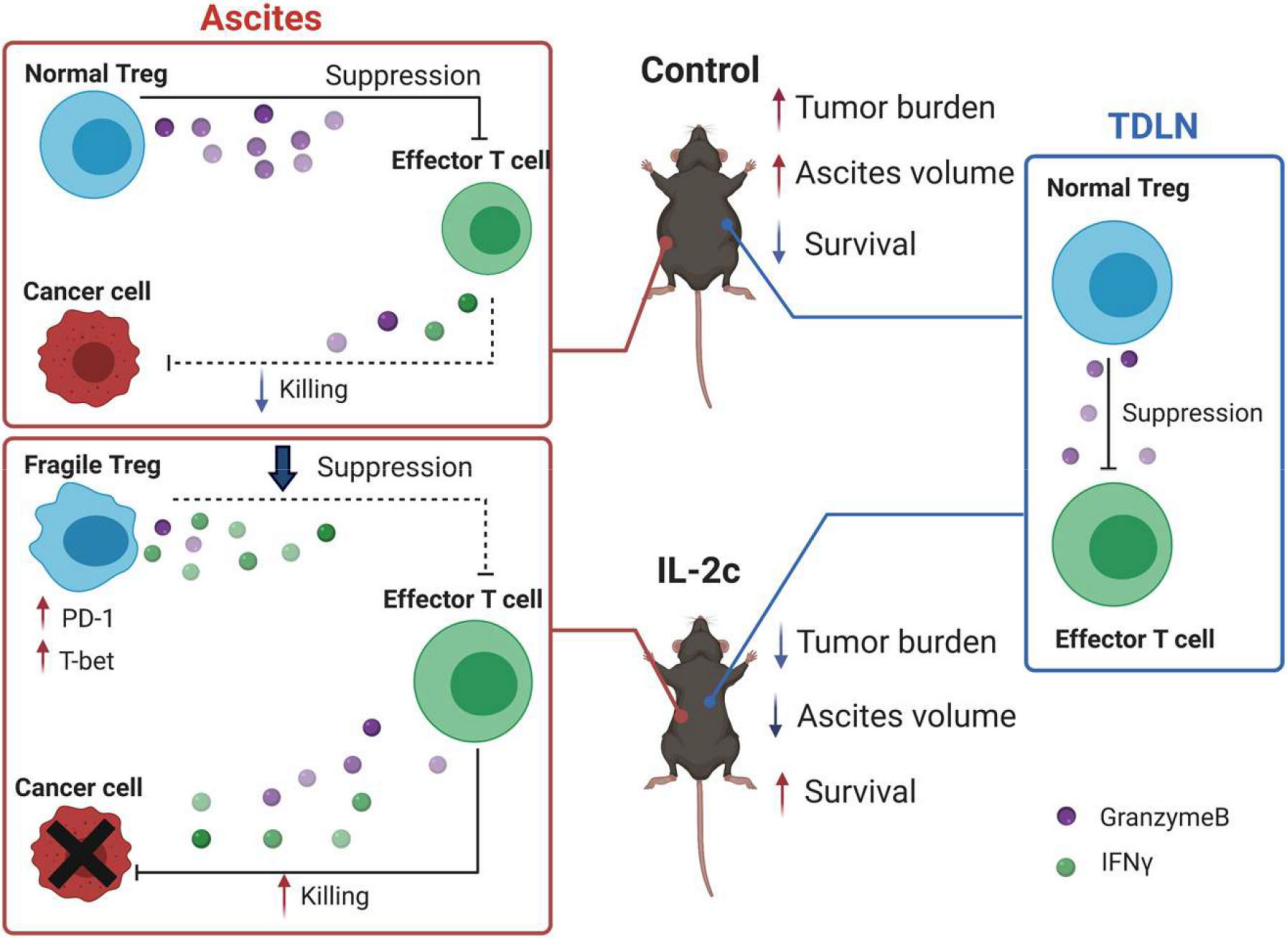
Graphical abstract of Drerup, Deng et al, Cancer Res. 2020 Nov 15;80(22):5063-5075. Major findings: 1) IL-2c inhibits growth of ID8agg mouse ovarian cancer and extends survival. 2) IL-2c increases effector T cell production of cytokines (e.g., IFNγ) and effector molecule (e.g., GranzymeB) in ascites.3) IL-2c decreases Treg production of effector molecule (e.g, GranzymeB) and inhibits Treg suppression in ascites, but not tumor draining lymph nodes. 4) IL-2c induces a fragile Treg phenotype in ascites, but not tumor draining lymph nodes. IL-2c synergizes with αPD-L1.

Recently, we reported beneficial treatment effects of IL-2c in a mouse ovarian cancer model [32] (major findings summarized in Figure 2), a significant advance in ovarian cancer (OC) immunotherapy for several reasons. OC is the deadliest gynecological cancer, leading to an estimated 13,700 deaths in the United States in 2021 [33]. It should respond to immunotherapies since intratumoral T cell infiltration predicts improved OC patient survival [34] which we showed is reduced by intratumoral Tregs [35]. However, decades of OC immunotherapy trials done by outstanding groups have been largely disappointing [36], which could be in part due to OC’s relatively low mutation burden compared to other tumors [36].

We found IL-2c is highly efficacious in the aggressive and poorly-responsive ID8agg mouse ovarian cancer line that we developed, including being refractory to immune checkpoint blockade (ICB) with α-programmed death ligand 1 (PD-L1). Consistent with known IL-2c effects, IL-2c promoted the activation of effector CD8^+^ T cells and CD4^+^ non-Treg cells and increased production of antitumor cytokines including IFNγ and TNFα by these cells in ID8agg challenge. Surprisingly however, IL-2c also expanded the number of Tregs thus reducing the CD8^+^ T cell/Treg ratio specially in ascites, an effect not previously reported with this approach, which could reflect the OC TME or OC cells themselves. Further investigation revealed that IL-2c treatment impaired Treg suppressive function in a TME-specific manner as Tregs from tumor draining lymph nodes (TDLN) suppressed normally.

Although the half-life of IL-2c is around 24 hours *in vivo* [11], we observed significant tumor-inhibitory effects as long as three weeks after the last dose of IL-2c. These data suggest induction of immune memory, evident by observed increases in central and effector memory CD8^+^ T cells after IL-2c treatment. Because IL-2c can increase TILs and tumors with pre-existing T-cell infiltration respond well to ICB [37], we explored combination of IL-2c and αPD-L1 therapy and found a synergistic effect of IL-2c plus αPD-L1 that was superior to either agent alone, in both (no t)ID8agg OC and B16 melanoma model. Moreover, mice that recovered from primary ID8agg challenge after combination therapy were resistant to subsequent tumor challenge, confirming induction of protective immune memory. Thus, IL-2c can work through simultaneous targeting of distinct immune pathways with effects that include: 1) activating effector T cells and promoting their antitumor cytokine production, 2) inhibiting immune-suppressive Tregs, and 3) inducing durable, protective immune memory.

**Figure 3:**
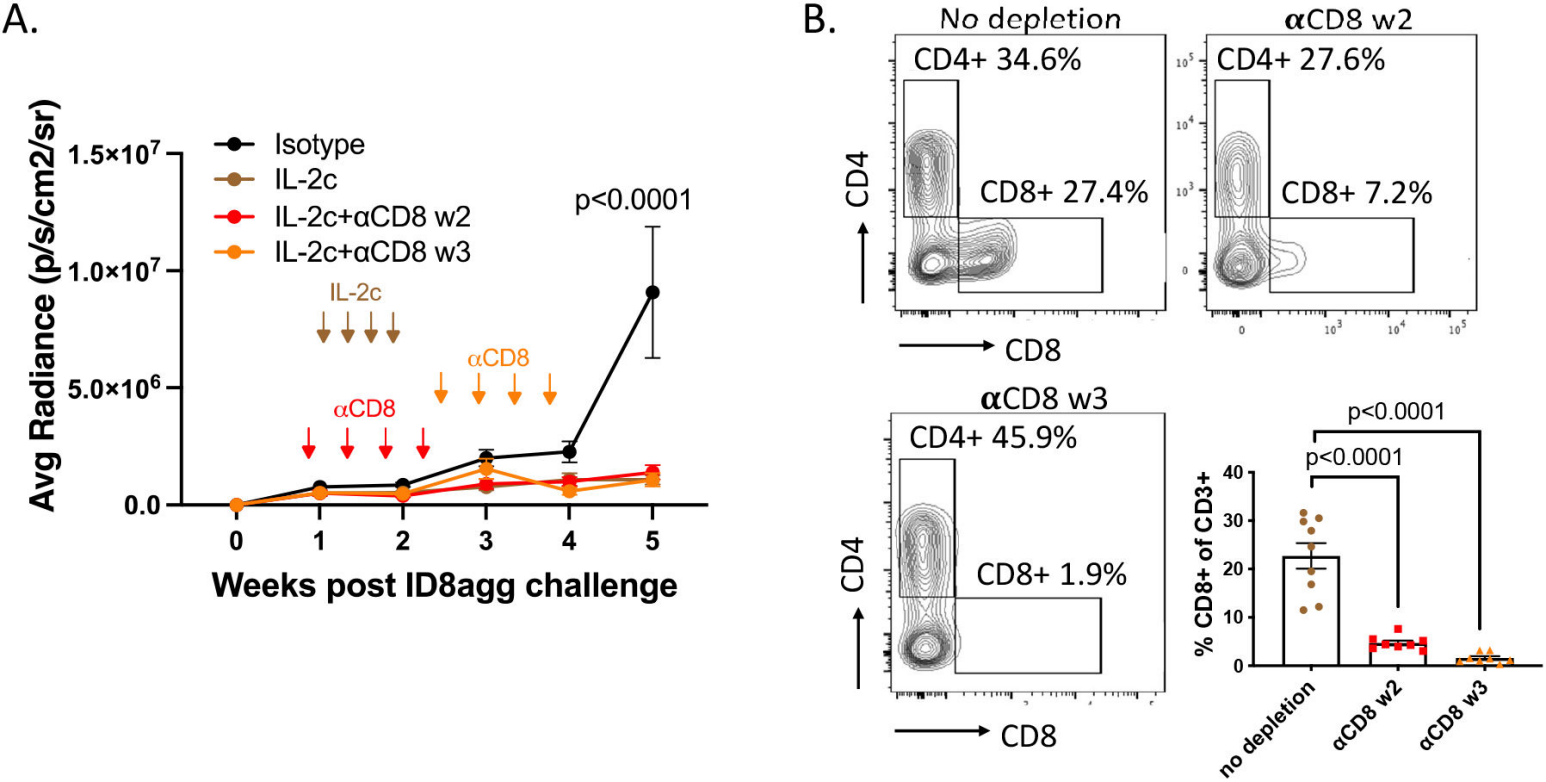
CD8^+^ conventional T cells are not required for the treatment efficacy of IL-2c. **A.** ID8agg tumors measured by luciferase as described [30]. Arrows indicate treatment of IL-2c or CD8 depleting antibody. N=8-9 per group, p value determined by two-way ANOVA. **B.** Flow analysis of tumor harvested on day 36 post ID8agg challenge. Contour plots are derived from CD3^+^ TILs. P value determined by one-way ANOVA.

In recent work, we performed additional exploration into the specific immune population(s) responsible for IL-2c efficacy in ID8agg tumors. CD8^+^ T cells are the primary target populations of IL-2c and are required for successful IL-2c treatment in several tumor models [9,14]. However, we found IL-2c was efficacious in ID8agg tumors following antibody-mediated CD8^+^ T cell depletion *in vivo* depletion. We tested two depletion timepoints, during the course of IL-2c treatment starting in week 2 or a week post the last dose of IL-2c (week 3), both of which significantly depleted CD8^+^ TILs for the duration of the experiment (Figure 3B) and did not affect IL-2c efficacy (Figure 3A). This result was striking because despite IL-2c mediated activation of CD8^+^ TILs in other models (Table 1), they were dispensable for IL-2c efficacy here. We also recently published that in orthotopic MB49 mouse bladder cancer, CD8 T^+^ cells were not required for IL-2c efficacy, which instead relied on δ T cells [31]. However, IL-2c treated ID8agg tumors effectively in γδ T cell-deficient mice [31], consistent with TME-specific IL-2c effects.

## IL-2c Induces TME Treg Fragility Independent of CD8+ and δ T cells

**Figure 4:**
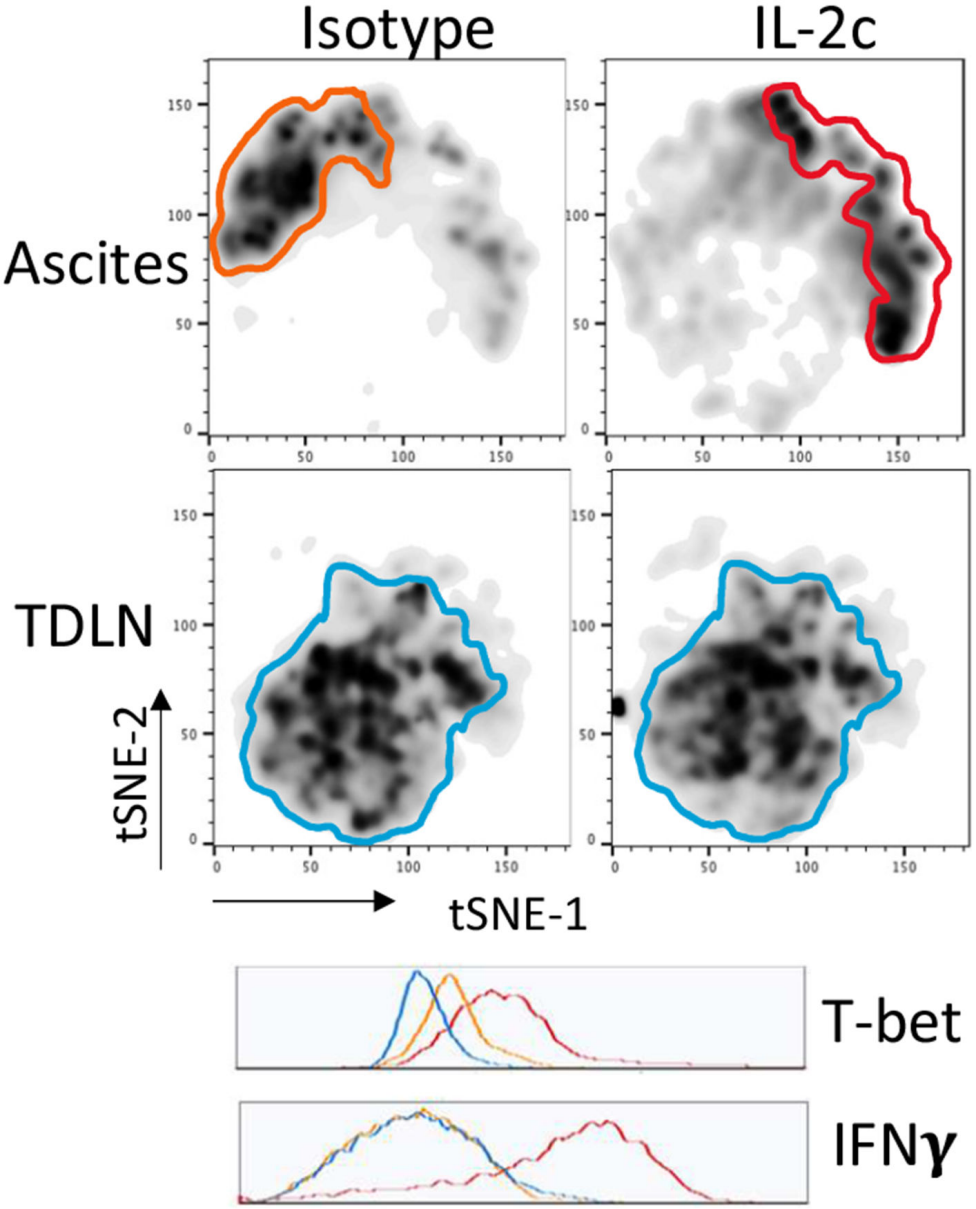
tSNE analysis of ascites Tregs in ascites and tumor draining lymph nodes from isotype or IL-2c treated mice challenged with ID8agg. Flow cytometry analysis performed three weeks post last IL-2c as described [30]. Density plots show highly different Treg phenotypes among ascites and tumor draining lymph nodes from isotype and IL-2c treated mice challenged with ID8agg. IL-2c treated ascites Tregs (red) exhibited increased expression of fragility markers including T-bet and IFNγ compared to isotype treated ascites Tregs (orange), among other changes. Tregs from tumor draining lymph nodes of two groups of mice are very similar to each other (blue).

Tregs are a major immune-suppressive population in the TME and are associated with worse outcomes in OC patients [35]. Tregs also play a crucial role in the development of ID8agg tumors as we previously showed that depleting Tregs completely abolished ID8agg tumor progression [32]. Strikingly, fragile Tregs were first described by the Vignali group and are characterized by maintained FoxP3 expression with loss of suppressive function and decreased production of effector molecules including granzyme B, versus an unstable Treg phenotype where Tregs lose their FoxP3 expression [38]. Fragile Tregs also express transcription factor T-bet and produce cytokines that are uncharacteristic of normal Tregs [39], including IFNγ. Treg fragility can also contribute to ICB treatment efficacy [38,39] which could be another mechanism of IL-2c synergy with αPD-L1. We found that Tregs displayed a fragile phenotype induced by IL-2c, which helps explain IL-2c efficacy despite increasing Treg numbers in the OC TME. We performed *t*-distributed stochastic neighbor embedding (*t*SNE) analysis of Tregs in the OC TME and TDLN and found that Tregs in TDLN treated with control or IL-2c have a similar immune phenotype, whereas IL-2c-treated Tregs in the ascites acquired the fragile phenotype (Figure 4). By contrast, IL-2c did not induce Treg fragility in the bladder in MB49 bladder cancer, consistent with TME-specific fragility induction.

**Figure 5:**
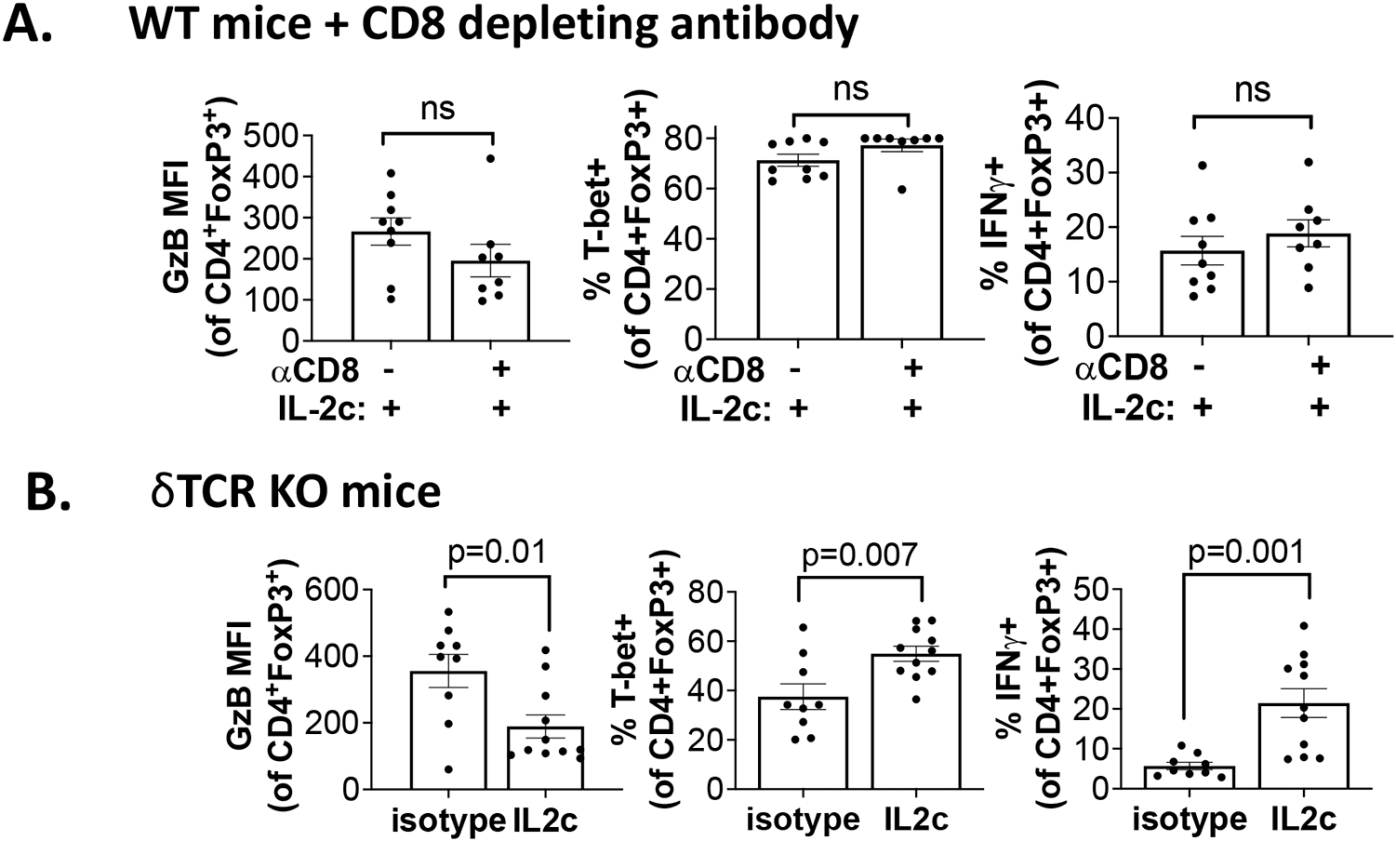
CD8^+^ conventional T cells and γδ T cells do not contribute to IL-2c induced Treg fragility. Wild type mice treated with CD8 depleting antibody (as in Figure 3). **(A)** or δTCR KO mice **(B)** were challenged with ID8agg and treated with IL-2c as described [30]. Ascites Treg phenotype by flow cytometry three weeks after final IL-2c. GzB: GranzymeB; P values, unpaired Student’s t-test.

We further explored mechanisms for IL-2c-induced Treg fragility. We observed that IL-2c-induced fragility can be detected three weeks after the final dose of IL-2c, but not one week after the final dose [32], and is likely indirect from CD25 signals. *Ex vivo* treatment of Tregs with IL-2c does not fully recapitulate the *in vivo* fragile phenotype [32], suggesting IL-2c cooperates with another factor or population in the OC TME to induce Treg fragility, or could work during Treg differentiation, which could account for the long time needed for *in vivo* fragility induction. IFNγ is a potent Treg fragility inducer [38] and IL-2c promotes IFNγ production in effector T cells. We assessed major IFNγ-producing population effects on Treg fragility induction, including CD8^+^ T cells and γδ T cells. Antibody-mediated CD8^+^ T cell depletion did not rescue the Treg fragility phenotype in ascites (Figure 5A), and fragile Tregs with increased T-bet, and IFNγ expression were also present in ascites of γδ T cell deficient mice challenged with ID8agg tumors (Figure 5B) excluding δ T cells and making CD8^+^ T cells unlikely mediators in IL-2c induced Treg fragility. Future studies will test NK cell, innate lymphoid cell and fragile Treg interferon-γ in IL-2c induced Treg fragility and assess other tumors and TME.

## Conclusions and Future Directions

Our published studies [31,32] and new data shown here highlight several important mechanisms that could be unique to the OC TME and serve as important contrasts to other studies using the same IL-2c or similar CD122-biased modified IL-2. First, conventional CD8^+^ T cells are not required for the treatment efficacy of IL-2c in ID8agg OC, despite IL-2c-mediated promotion of IL-2 signaling and antitumor cytokines production in intratumoral CD8^+^ T cells. In this specific TME, IL-2c preferentially activates other immune populations that play a more crucial role than CD8^+^ T cells in treatment efficacy. Absence of OC TME CD8^+^ T cells is not seen and thus agents such as αPD-L1 that do activate OC TME CD8+ T cells can synergize with IL-2c as we reported. Other IL-2c activated populations including NK cells or other members of the innate immunity could be preferentially activated over CD8^+^ T cells or insufficient CD8^+^ T cells could induce compensatory activation of other cell populations. Understanding the preferential and distinct IL-2 signaling and activation of varied immune populations in specific TMEs and/or tumors is essential for comparing differences in IL-2 targeting agent mechanisms between various tumors and anatomic locations, and to define mechanisms for agents that can cooperate clinically with directed IL-2 agents, which to date is little studied. Second, IL-2c inhibits intratumoral Treg function by inducing a fragile phenotype at least in some tumors and environments. CD122-selective IL-2 agents are expected to avoid activation and proliferation of Tregs and most previous studies in other models observed an increased CD8^+^ T cell/Treg ratio [8,14,22,40] and sometimes even Treg depletion [19]. However, we found increased number of Tregs and fragility in ascites after IL-2c treatment. Studies are warranted to identify fragility mediators, define in what other cancer models or human cancers fragile Tregs can be induced and to understand which agents best combine with this effect for treatment efficacy.

Currently, many modified IL-2 agents are in varied stages of preclinical and clinical development [23] (and Table 1), yet a detailed understanding of the immune landscape during treatment is lacking in most cases. Our prior and ongoing investigations have uncovered unexpected yet useful immune consequences of IL-2c signaling in the OC TME, stressing the importance of understanding the biology and immunology of distinct IL-2 agents to advance our understanding of IL-2 and IL-2 receptor biology, and optimal therapeutic uses. We also shed light on OC-specific TME phenotypes that potentially help explain its unresponsiveness to current immunotherapies, which should facilitate further design of targeted immunotherapies for OC patients.

## Figures and Tables

**Table1:** 

Author	Year	Agent Tested	Immune cell activation *invitro*	Immune cell activation in non- tumor bearing mice	*in vivo* efficacy	Combination therapy	Population responsible for efficacy	Immune change in TME	Ref
Boyman et al	2006	IL-2/mAb complex	Mouse & human Teff proliferation						7
Kamimura et al	2006	antiIL-2 mAb + IL-2 cDNA		Expansion of CD8, NK cells, decrease Tregs	B16 lung metastasis				24
Verdeil etal	2008	IL-2/mAb complex	Mouse & human Teff proliferation, cytokine production		pancreatic model	vaccination		CD8 expansion, pSTAT5, effector molecule cytokine production	8
Jin et al	2008	IL-2/mAb complex		Expansion of DC, NK, CD8 in spleen, increased perforin and GB for NK cells	B16 lung metastasis		NK cells required, but not DCs		30
Tomala etal	2009	IL-2/mAb complex		Both S4B6 and JES6-1 are able to activate CD8 T cells; Only S4B6 activate NK cells	B16 SQ		CD8 and NK cells required		9
					BCL1 leukemia		CD8 required		
Letourneau et al	2010	IL-2/mAb complex		CD8+ t cell proliferation					11
Krieg et al	2010	IL-2/mAb complex		Expansion of CD8, NK and Tregs	B16 SQ, B16 lung				10
Redmond et al	2012	IL-2/mAb complex			MCA-205 sarcoma	OX40 agonist Ab	T cells required		14
					TRAMP-C1-mOVA prostate		[doesn't respond to IL-2c alone]	Expansion of OT-1 CD8 (ki67,GB, KLRG1)	
Cho et al	2012	IL-2/mAb complex			B16 SQ	vaccination, adoptive T cell transfer, aPD-L1			21
Raeber etal	2020	IL-2/mAb complex	No cDC activation *in vitro*	cDC expansion *in vivo* through IL-2 stimulated ILCs	B16 SQ, Braf melanoma		B16: DC is required	Expansion of cDC1 in the tumor	25
Drerup et al	2020	IL-2/mAb complex			ID8agg OC,B16 SQ	aPD-L1		Activation of tumor CD8+ and CD4+ non-Tregs with increased production of anti-tumor cytokines. Induce Treg fragility in local TME but not TDLN.	32
Reyes et al	2021	IL-2/mAb complex			MB49, MBT-2 orthotopic Bladder Cancer, MB49 metastatic lung	aPD-L1	MB49: CD8 not required; γδ T cells required	No activation of CD8+ seen inMB49 orthotopic. Significantly increased intratumoral NK and γδ T cell content, activation, and effector function	31
Gillies etal	2011	NHS-IL2LT IL-2 variant fusion with an antibody (NHS76)	mouse CD8, CD4 (proliferation)		LLC lung cancer		CD8 and NK cells required, CD4 not required		12
					NXS2 neuroblastoma liver metastasis				
Levin et al	2012	IL-2 superkine & IL-2/mAb complex	human NK cell pSTAT5 status (also in CD25−/−)	CD8^+^ expansion	B16 SQ; B16 lung; MC38,Lewis lung carcinoma				13
Carmenate et al	2013	human IL-2 mutein	Mouse NK, CD8 proliferation but not Tregs		MB16F0 melanoma lung metastasis		CD8 and NK cells required		15
					3LL-D122 mouse lung carcinoma				
Charych et al	2016	Bempegaldesleu kin NKTR-214 (pegylated IL-2)	mouse T cells pSTAT5		B16 SQ	aCTLA-4		Expansion of CD8, NK, decreased CD4, increased intratumoral CD8/Treg ratio	22
					EMT6 breast cancer, CT26 colon model				
Charych et al	2017	Bempegaldesleu kin: NKTR-214 (pegylated IL-2)		pSTAT5 on CD3+ in PB	MBT-2 (bladder), H22(liver), Pan02 (pancreatic)				18
Sockolosky et al	2018	orthoIL2	(CD8, Tregs)pSTAT5, proliferation		B16			Increased CD8IFNg production, PD-1 Tim3 expression	17
Sun et al	2019	Ab-sumIL2			B16	aPD-L1	CD8 required; NK, MDSC, CD4 not required	Increased CD8/Treg ratio, increased CXCR5+PD-1+ cells, increase PD-L1 on DCs	40
					MC38				
Sahin et al	2020	hIL-2/NARA1	pSTAT5 comparison Cytotoxic T cells, NK cell, Tregs	pSTAT5, Ki67, cell counts, for CD8, NK and Tregs	B16 (sQ and metastatic) LLC lung; 4T1breast		4T1: doesn't treat primary as single agent but reduces metastasis	hIL-2/NARA1: increase CD8, NK1.1, Treg; NARA1leukin: not Treg; lower exhausted T cells	26
Sharma et al	2020	Bempegaldesleu kin			B16, Colon (CT-26), ovarian (BR5FVB), bladder (MBT-2), liver (H22), pancreatic (Pan02), breast (EMT-6), lung (LLC),	aPD-1, vaccine			19
Hsu et al	2021	ProIL2			MC38, CT26,B16	aPD-L1		Increased CD8, decreased Foxp3, increased ratio	29
